# The RNA sequencing results revealed the expression of different genes and signaling pathways during chemotherapy resistance in peripheral T-cell lymphoma

**DOI:** 10.1186/s12920-024-01842-6

**Published:** 2024-03-11

**Authors:** Yunyi Lan, Wei Tao, Luyao Ma, Xiaoxiong Wang, Hongsheng Li, Yaxi Du, Ruijiao Yang, Shunxian Wu, Yingxin Ou, Xin Liu, Yunchao Huang, Yongchun Zhou

**Affiliations:** 1 Molecular Diagnostic Center, The Third Affiliated Hospital of Kunming Medical University, Kunming, China; 2 Department of Hematology, The Second Affiliated Hospital of Kunming Medical University, Kunming, China; 3 International Joint Laboratory On High Altitude Regional Cancer, Kunming, China; 4 Yunnan Key Laboratory of Lung Cancer Research, Kunming, China

**Keywords:** Peripheral T-cell lymphoma, Chemotherapy resistance, RNA sequencing, Differentially expressed genes, Signaling pathways

## Abstract

**Background:**

Peripheral T-cell lymphoma (PTCL) is a subtype of non-Hodgkin's lymphoma that occurs primarily at extranodal sites and is commonly treated using chemotherapy and radiotherapy. PTCL is more malignant than other lymphoid tumors, resulting in a poor prognosis.The 5-year recurrence rate remains high, and there is a lack of standard treatment for patients with relapse-resistant disease. However, the molecular mechanisms underlying the resistance of peripheral T-cell lymphoma cells to chemotherapeutic drugs, as well as identifying strategies to overcome drug resistance remains unclear. In this study, we aimed to identify pivotal genes and signaling pathways associated with chemotherapy resistance in PTCL.

**Methods:**

In this study, a total of 5 healthy controls and 7 clinical patients were enrolled; 4 patients were classified as chemotherapy sensitive, and 3 patients were classified as chemotherapy resistant. Peripheral blood samples were collected from each participant, and total RNA was extracted from the white blood cells. RNA sequencing was conducted on the Illumina HiSeq platform to obtain comprehensive gene expression profiles. Subsequently, the expression patterns of the DEGs associated with the most enriched signaling pathways, with a special focus on cancer-related genes, were validated using quantitative real-time polymerase chain reaction (qRT–PCR) in peripheral TCL patients.

**Results:**

RNA sequencing (RNA-seq) analysis revealed 4063 differentially expressed genes (DEGs) in peripheral T-cell lymphoma specimens from patients with chemotherapy resistance, of which 1128 were upregulated and 2935 were downregulated. Subsequent quantitative gene expression analysis confirmed a differential expression pattern in all the libraries, with 9 downregulated genes and 10 upregulated genes validated through quantitative real-time PCR in 6 clinical specimens from patients with chemotherapy resistance. KEGG pathway analysis revealed significant alterations in several pathways, with 6 downregulated pathways and 9 upregulated pathways enriched in the DEGs. Notably, the TNF signaling pathway, which is extensively regulated, was among the pathways that exhibited significant changes. These findings suggest that DEGs and the TNF signaling pathway may play crucial roles in chemotherapy resistance in peripheral T-cell lymphoma.

**Conclusion:**

Our study revealed that the expression of specific genes, including TNFRSF1B, TRADD2, and MAP3K7, may play an important role in chemotherapy resistance in peripheral T-cell lymphoma. Moreover, we identified the downregulation of the TNF signaling pathway, a crucial pathway involved in cell survival, death, and differentiation, as a potential contributor to the development of chemotherapy resistance in peripheral T-cell lymphoma. These findings provide valuable insights into the molecular mechanisms underlying chemotherapy resistance and highlight potential targets for overcoming treatment resistance in this challenging disease.

## Introduction

Peripheral T-cell lymphoma (PTCL) is a specific type of non-Hodgkin lymphoma characterized by the proliferation of mature T cells. This disease primarily manifests in extranodal sites such as the intestinal tract, skin, lymph nodes, spleen, and other tissues outside of the bone marrow. Common symptoms include fever, night sweats, weight loss, itching, and other systemic manifestations [1, 2]. The specific etiology of PTCL has not been determined and various risk factors have been identified for its development. These include viral infections such as Epstein–Barr virus and human T lymphocyte virus, exposure to radiation or certain chemicals, and abnormalities in the immune system [3].

The incidence of peripheral T-cell lymphoma (PTCL) varies across different regions, and PTCL is considered a relatively rare malignancy, accounting for approximately 10–15% of all non-Hodgkin lymphoma (NHL) cases [4]. However, in Asian countries such as China and Japan, the incidence of this disease is relatively high, whereas it is lower in western countries [5]. Aggressive subtypes of PTCL, such as extranodal nasal NK/T-cell lymphoma and cutaneous T-cell lymphoma, are characterized by rapid disease progression, short survival periods, and poor prognosis [6]. Overall, peripheral T-cell lymphomas have an unfavorable prognosis and necessitate intensive therapeutic approaches [7].

The management of PTCL involves a multimodal treatment approach that involves combination of chemotherapy and radiotherapy [8]. Chemotherapy is currently the standard treatment for hematopoietic tumors such as lymphoma; however, the development of chemotherapeutic drug resistance remains a major obstacle to achieving successful treatment outcomes and preventing tumor recurrence. Therefore, investigating the molecular mechanisms underlying the resistance of peripheral T-cell lymphoma cells to chemotherapeutic drugs, as well as identifying strategies to overcome drug resistance, has emerged as a key research area in the field of chemotherapy for hematopoietic tumors [9]. Clinical investigations have demonstrated the efficacy of gemcitabine-based chemotherapy regimens in the treatment of PTCL. However, studies have reported the generation of gemcitabine-resistant human peripheral T-cell lymphoma cell lines, as well as investigations into the mechanisms underlying drug resistance [10].

Over the past few years, genetic analyses of peripheral T-cell lymphoma (PTCL) have advanced our understanding of the pathogenesis of this malignant tumor. Activating mutations in the NF-κB, Notch, JAK/STAT3, RHOA, and PI3K/AKT signaling pathways have been found to play crucial roles in the pathogenesis of PTCL. For instance, NF-κB is involved in proliferative signaling in various lymphoid malignancies, and although further investigation is needed, gene expression profiling studies have shown increased expression of NF-κB-related genes in PTCL, and the induction of apoptosis in PTCL cells by NF-κB inhibitors supports the notion that this pathway plays a significant role in PTCL. Moreover, accumulating evidence suggests the involvement of viral components in multidrug chemotherapy resistance in lymphoma cells, with some mechanisms potentially mediated through cancer-causing viruses altering disease signaling pathways [11].

Transcriptome sequencing, performed using the Illumina sequencing platform, involves the comprehensive analysis of all RNA molecules transcribed by a specific cell or tissue at a given time, including both coding and noncoding mRNAs. This technique provides valuable insights into gene function and structure and plays a crucial role in understanding organism development and the pathogenesis of diseases [12]. Therefore, our study aimed to elucidate the molecular mechanisms underlying chemotherapeutic drug resistance in peripheral T-cell lymphoma cells through transcriptome analysis. With this approach, we hope to identify novel molecular targets and dysregulated molecules within the carcinogenic pathways, offering potential insights for therapeutic interventions.

## Materials and methods

### Selection of patients and sample preparation

Eligible participants for this study included individuals aged aged over 18 years who had a confirmed histological diagnosis of peripheral T-cell lymphoma, as per the World Health Organization (WHO) classification of lymphoid neoplasms. Patients were required to have documented relapse or disease progression following prior chemotherapy treatment with gemcitabine, as indicated in Table 1. The table presented within the study consisted of different cohorts, including the discovery and validation cohorts. The control group consisted of healthy control samples, while the sensitivity group comprised samples from patients exhibiting chemotherapy sensitivity. Conversely, the experimental group included samples from patients with chemotherapy resistance. The allocation of samples into these respective groups was performed based on predetermined criteria and classification.

**Table 1:** Characteristics of the samples in the pilot study (*n* = 12)

Discovery cohorts	Sample Name	Age (year)	Gender	Proportion of primary lymphocytes	Histopathological characterization	Drug regimens
Control	Sample 1	37	Male	-	-	-
	Sample 2	38	Male	-	-	-
	Sample 3	28	Female	-	-	-
	Sample 4	25	Female	-	-	-
	Sample 5	34	Female	-	-	-
Sensitivity	Sensitivity_1	59	Male	13%	NK/T lymphoblastic lymphoma, stage IV group B, IPI 3 score	P-GELOX (gemcitabine 1.4 mg, d1, 8; Pemendon 3750 d1; Oxalipol 150 mg d1)
	Sensitivity_2	59	Male	-	Extranodal NK/T cell lymphoma, nasal type, Stage IV Group A, IPI 2 score	P-GELOX (gemcitabine 1.8 g, d1, 8; Pemendon 3750 d1; Oxalipol 180 mg d1)
	Sensitivity_3	26	Male	0.5%	Primary cutaneous peripheral T-cell lymphoma	P-Gemox(Pemaspartase 3750 u d1, gemcitabine 1.6 g d1, d8, oxaliplatin 150 mg d1)
	Sensitivity_4	43	Female	2%	Primary cutaneous peripheral T-cell lymphoma	GDP + etoposide (gemcitabine 1.3 g, d1, 8; Dexamethasone 40 mg d1-4; Cisplatin 100 mg d1; Etoposide 80 mg d1-4)
Experimental	Experimental_1	47	Male	-	NK/T cell lymphoma stage II Group A, IPI 1 Score	P-Gemox(Pemaspartase 3750 u d1, gemcitabine 1.6 g d1, d8, oxaliplatin 150 mg d1)
	Experimental_2	62	Male	-	Peripheral T cell lymphoma stage III Group B, IPI 1 Score	GDP (Gemcitabine 1.6 g, d1, 8; Dexamethasone 40 mg d1-4; Cisplatin 120 mg d1)
	Experimental_3	51	Male	25%	T-lymphoblastic lymphoma, stage III Group B, IPI 1 Score	Sidanidine + GDP (Sidanidine 30 mg, d1, 4, 8, 11; Dexamethasone 40 mg, d1-4; Gemcitabine 1.6 g, d1, 1.2 g, d8; Cisplatin 120 mg, d1)
Sensitivity	Sensitivity_5	54	Male	-	T-lymphoblastic lymphoma stage I, low risk group. (No IPI score)	COP tumor reduction and BFM-90 treatment were performed 4 times
	Sensitivity_6	70	Male	-	Extranodal NK/T cell lymphoma, nasal type, Stage IIB, IPI2 score, Low risk group	P-GELOX (gemcitabine 1.8 g, d1, 8; Pemendon 3750 d1; Oxalipol 180 mg d1) and radiotherapy
	Sensitivity_7	25	Female	-	Acute leukemia	MA scheme treatment were performed
Experimental	Experimental_4	16	Male	-	Stage III T-lymphoblastic lymphoma (mediastinum, neck, supraclavicular lymph nodes)	VDLP scheme, CAM scheme were performed twice, M scheme treatment were performed 3 times
	Experimental_5	55	Male	-	Peripheral T cell lymphoma, non-specific type, stage IVB, IPI 4 score, high risk group	CHOP scheme were performed twice, ESHAP scheme, GDP and sidarbenamide scheme treatment
	Experimental_6	60	Female	-	T-lymphoblastic lymphoma stage III, low risk group. (No IPI score)	VDLP + CAM scheme, M scheme were performed 4 times and VDLP scheme treatment

Peripheral blood or bone marrow blood samples were taken in 2020 and 2021 at the Department of Hematology of the Second and the Third Affiliated Hospital of Kunming Medical University, Yunnan, China. The Ethics Committee of Kunming Medical University approved this research. After sample collection, the leukocytes were immediately separated from each sample and stored at − 80 °C until further processing and sequencing.

### Ritchie Giemsa staining

The anticoagulant EDTA was used to absorb 5–7 μl of peripheral blood via a capillary pipette, after which the blood was dropped on a glass slide. Apply Garry's Giemsa A solution to the smear, and let the solution cover the whole specimen for 1 min. Then, Rei's Jemsa B solution was added to liquid A (the amount of drip was 2–3 times that of liquid A), after which the mixture was blown through the mouth or earball to make the liquid surface ripple so that the two liquids were fully mixed and stained for 3–10 min. Then, the slides were washed and dried, after which the blood cells were examined via microscopy.

### RNA quantification and RNA library construction

RNA was extracted from leukocytes with an RNeasy® Mini Kit (Qiagen, Duesseldorf, Germany, cat# 74104), and a Nanodrop and an RNA Nano 6000 Assay Kit from a Bioanalyzer 2100 system (Agilent Technologies, CA, USA) were used to evaluate the quantity and stability of the RNA.

The initial RNA used for library construction was total RNA, which was enriched with oligo(dT) magnetic beads for poly(A) tail mRNA, and then randomly disrupted by divalent cations in fragmentation buffer. With the fragments of mRNA serving as templates and random oligonucleotides used as primers, the M-MuLV reverse transcriptase system was used to synthesize the first cDNA strand. RNaseH degrades RNA strands, and DNA polymerase I synthesizes the second strand of cDNA with dNTPs. Following terminal repair of the purified double-stranded cDNA, a tail was added, and the sequence was joined. AMPure XP beads were subsequently employed to screen cDNA approximately 370–420 bp in length for PCR amplification, after which the beads were reused to purify PCR products and obtain the final library.

After constructing the library, a Qubit 2.0 fluorometer was used for initial quantification, and the library was diluted to 1.5 ng/µl. Subsequently, an Agilent 2100 bioanalyzer was utilized to determine the insert size of the library.

### Computer sequencing

After a thorough library review, Illumina platform sequencing was employed to amalgamate and sequence multiple libraries, resulting in 150 bp paired-end reads that were in accordance with the desired concentration and data volume. The fundamental principle of sequencing is the sequencing by synthesis(SBS).

Four fluorescently labeled dNTPs, DNA polymerase and joint primers were added to the sequencing flow cell for amplification. Each cluster was fluorescently labeled because it extended complementary chains. dNTP can emit the corresponding fluorescence, and the optical signal is transformed into a sequencing peak to obtain sequence information.

### Data analysis

The sequencer’s measured image data were converted into sequence reads. Initially, fastq formatted raw reads were processed through internal Perl scripts. Clean reads were subsequently obtained by eliminating reads with adapters, N bases, and low-quality reads. Simultaneously, the Q20, Q30, and GC content of the clean data were calculated.

We employed fastp software (version 0.23.1) for quality control. The specific parameters used were as follows: qualified_quality_phred set to 5, unqualified_percent_limit set to 50, n_base_limit set to 15, min_trim_length set to 10, overlap_len_require set to 30, overlap_diff_limit set to 1, overlap_diff_percent_limit set to 10, length_required set to 150, and length_limit set to 150. Additionally, a trim_poly_g step was incorporated in the quality control process.

To obtain the necessary reference genome and gene model annotation files, we obtained them directly from the appropriate genome website [13]. These files were downloaded and utilized for subsequent analysis. Next, HISAT2 software was used to align the clean reads to the reference genome. This alignment process allowed for the determination of the exact position of the reads on the reference genome, providing valuable information on their genomic location. This step was conducted to facilitate further analysis and interpretation. Following the alignment, FeatureCounts software (version 1.5.0-p3) was used to count the number of reads associated with each gene. This step involved tallying the reads that uniquely mapped to each gene within the alignment data. Subsequently, the fragments per kilobase of transcript per million mapped reads (FPKM) values for each gene were calculated. The FPKM values were determined based on the length of the gene and the number of reads mapped to it [14].

For the differential expression analysis between two groups, we utilized the DESeq2 R package (version 1.20.0) [15]. To control for the false discovery rate, the p values were adjusted using the Benjamini–Hochberg method. The threshold for determining significantly differentially expressed genes (DEGs) was set at a padj value of less than or equal to 0.05 and a log2-fold change absolute value greater than or equal to 1. To validate the accuracy of the identified DEGs, a quantitative real-time PCR comparison was performed on six clinical individuals with peripheral T-cell lymphoma. Specifically, nine downregulated DEGs (AKT1, NFKBIA, TRADD, MAP2K1, MAP2K6, MAP3K7, PIK3CD, TRAF1, and TNFRSF1B) and ten upregulated DEGs (CAMTA1, HIST1H3B, ARHGEF12, PBX1, HIST1H4I, TAL1, YWHAE, ACVR1, MAX, and GNAS) were confirmed through this experimental validation.

To conduct a Gene Ontology (GO) enrichment analysis of the DEGs, we utilized the ClusterProfiler R package (version 3.8.1) [16]. This analysis took into account the bias that may arise due to differences in gene length. GO terms with a corrected *p* value less than 0.05 were considered to indicate significant enrichment of the DEGs. To assess the statistical enrichment of DEGs according to the Kyoto Encyclopedia of Genes and Genomes (KEGG) (Kyto reactome or Disease Ontology) pathways, we again used the ClusterProfiler R package (version 3.8.1). The Reactome database provides comprehensive pathway information, while the DO database describes the functions of human genes and diseases. Significantly enriched pathways were identified based on a corrected *p* value threshold of less than 0.05.

To assess whether a predefined gene set exhibits significant differences between two biological states, the gene set enrichment analysis (GSEA) computational approach can be employed. This analysis takes into account even subtle changes in gene expression. In our study, we utilized a variety of data sets, including Gene Ontology (GO), Kyoto Encyclopedia of Genes and Genomes (KEGG), Reactome, DO, and DisGeNET data sets, to perform GSEA independently. These data sets provided valuable information on gene function, biological pathways, and disease associations. By performing GSEA using these data sets, we were able to identify gene sets that were significantly enriched and showed notable differences between the two biological states under investigation. This computational approach enabled us to gain insights into the functional implications and potential disease relevance of the differentially expressed genes.

### Statistical analyses

The statistical analysis was performed using GraphPad Prism 5 software (GraphPad Software, La Jolla, CA, USA). To assess the differences between multiple groups, one-way analysis of variance (ANOVA) followed by Dunnett's multiple comparison test was used. The results are presented as the mean ± standard deviation (SD). For comparisons between two groups, an unpaired Student's t test was used. A *p* value of less than 0.05 (*) was considered to indicate statistical significance, while *p* values of less than 0.01 (**) and less than 0.001 (***) were considered to indicate high significance.

## Results

### Histopathological characterization

**Fig. 1:**
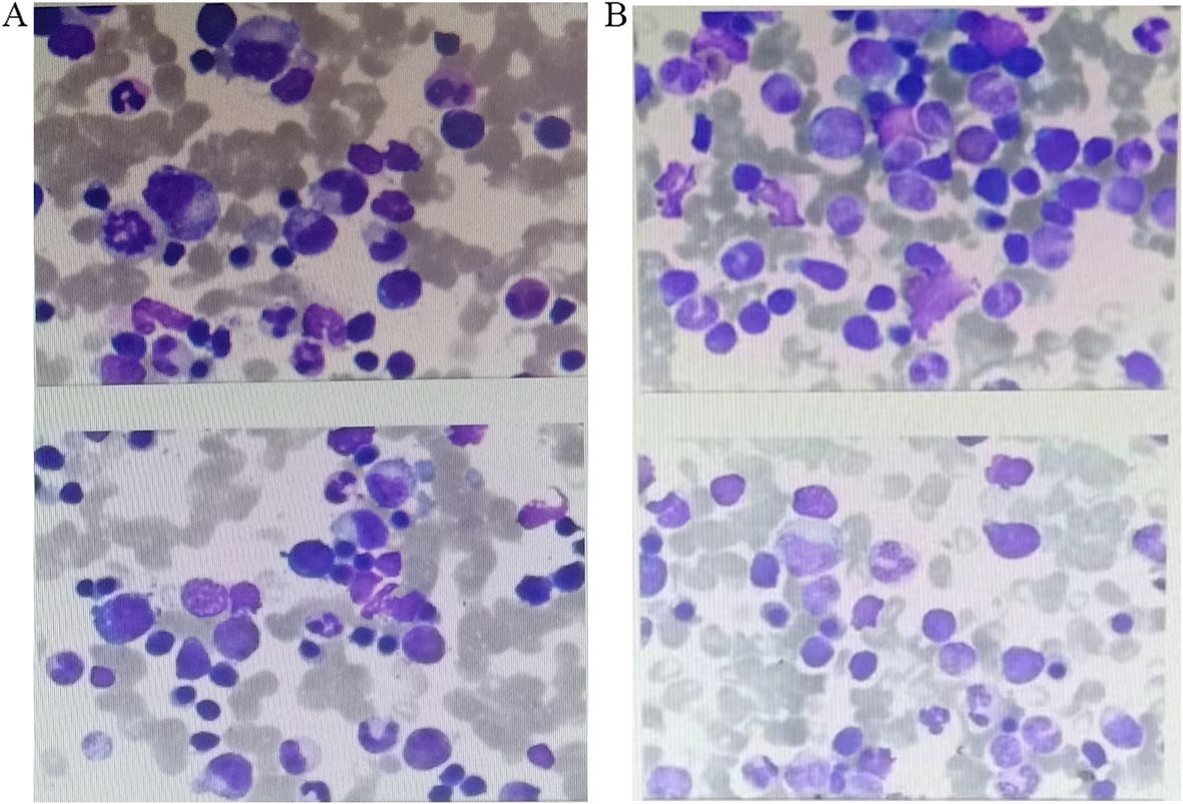
Histopathological characterization between chemotherapy sensitivity and chemotherapy resistance PTCL samples. **A** The Bone marrow morphology of the chemotherapy sensitivity sample: Sensitivity_4. **B** Bone marrow morphology of the chemotherapy resistance sample: Experimental_3

To analyze the presence of tumor cells in the blood samples, a Richs–Giemsa mixed staining method was used. This staining technique allowed for the detection of primitive blood cells in patient samples. As depicted in Fig. 1, the results clearly demonstrated the presence of these primitive blood cells in the blood samples. These findings indicate that the blood samples met the necessary criteria and were suitable for further RNA sequencing experiments.

### Sequencing and transcriptome data

**Fig. 2:**
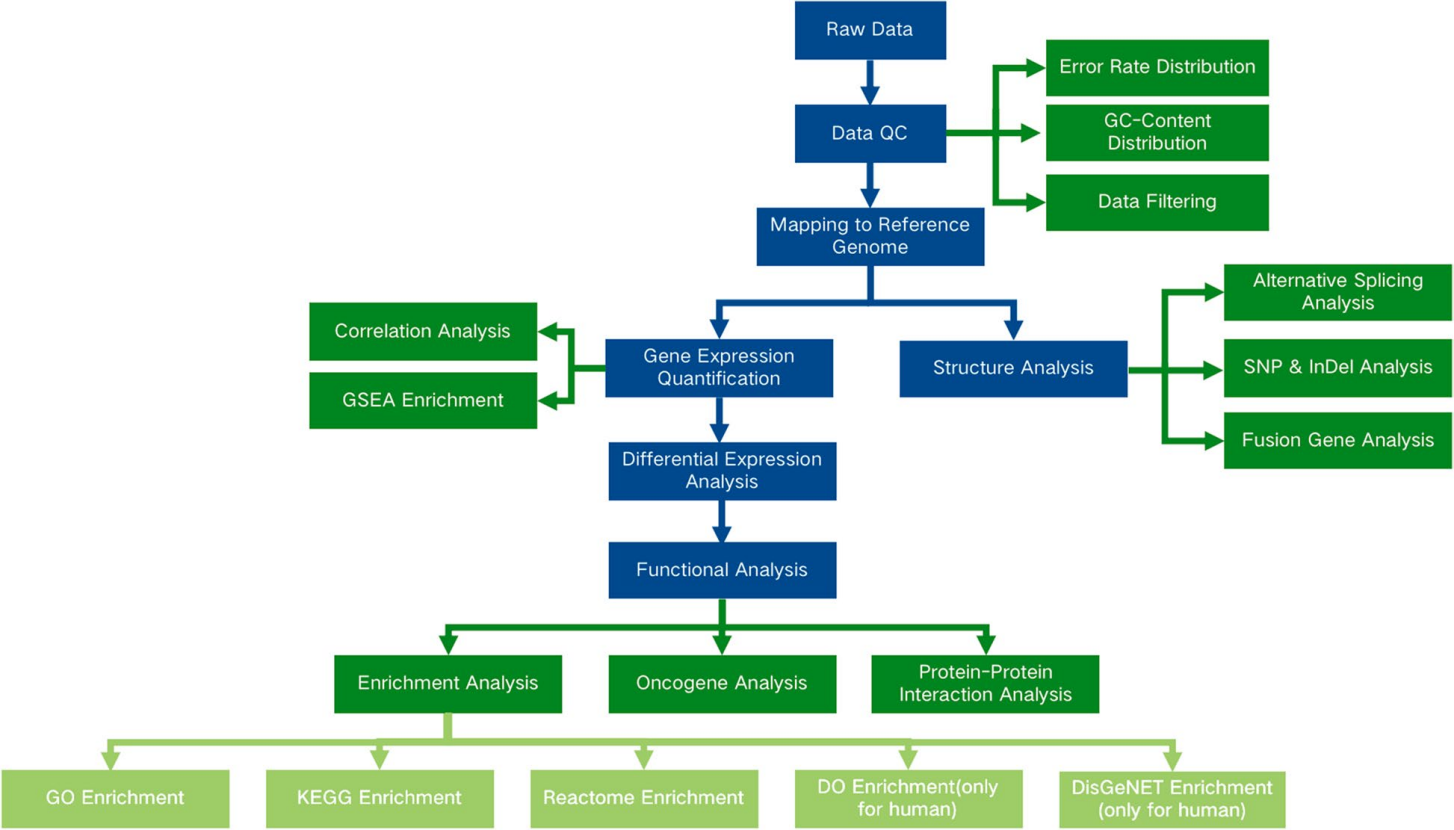
RNA Sequencing analysis process

The use of RNA-seq in the nucleus lies in the examination of gene expression disparities. Statistical techniques are used to detect differences in gene expression between two or more conditions, uncovering particular genes associated with those conditions and further examining the biological importance of these genes. The examination process involved quality control, comparison, quantitative analysis, significance analysis of differences, functional enrichment, and other connections. In addition, variable splicing, mutation site and fusion gene prediction data are also important for analyzing RNA-seq data. Moreover, according to different research needs, we performed personalized transcriptome analysis, such as gene coexpression network construction (WGCNA) and somatic mutation detection. The information analysis process is shown in Fig. 2.

**Fig. 3:**
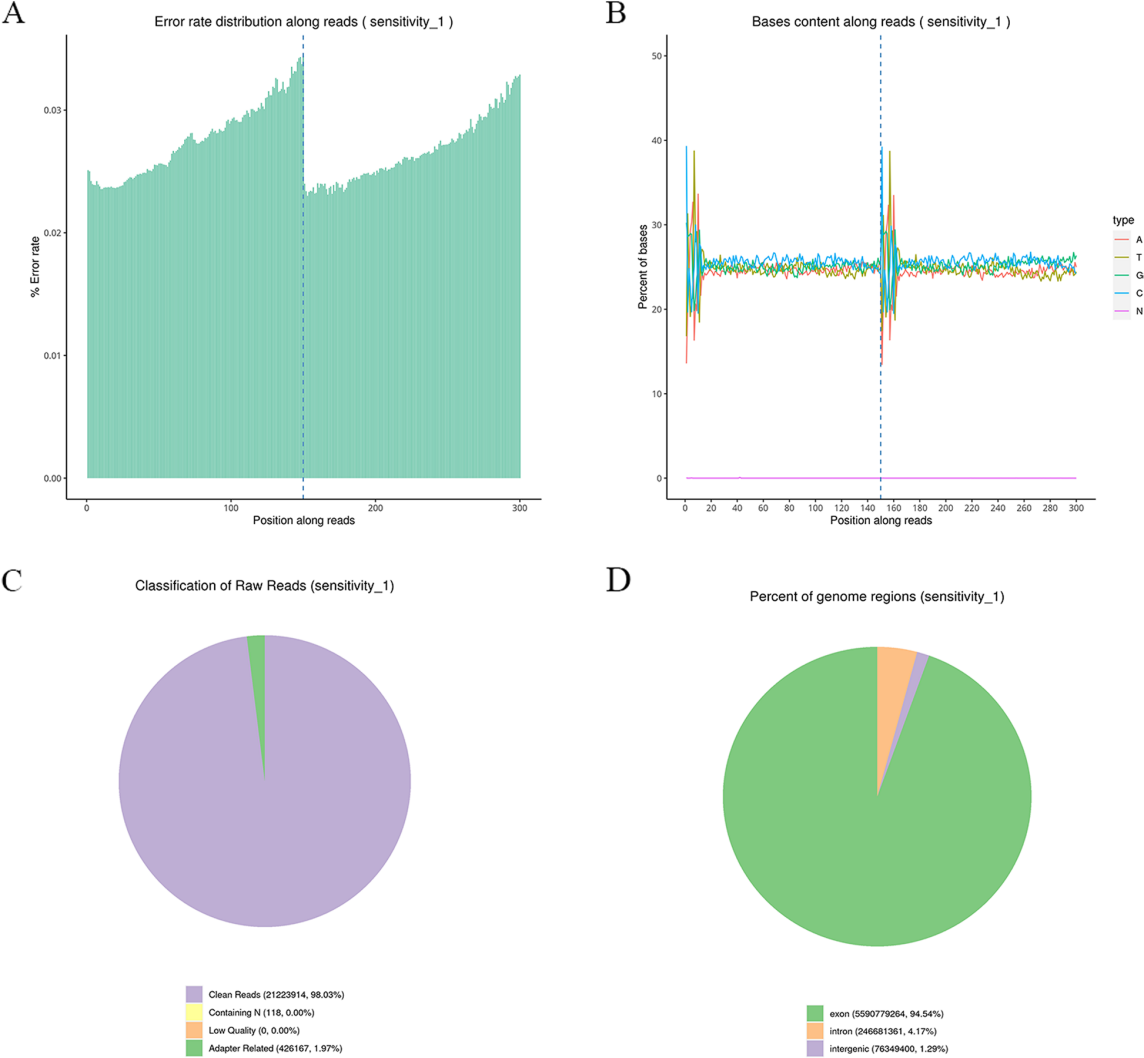
Data quality control of the RNA sequencing sample. **A**-**D** The figures show the error rate distribution, GC content distribution, sample sequencing data filtering and reference genome alignment of the RNA sequencing sample

The sequencing process itself has the possibility of machine errors. A quality check of the sequencing data's error rate distribution was performed. The sequencing quality value of each base in the sequence information is stored in the fastq file. Figure 3A shows the sample sequencing error rate distribution, with e representing the sequencing error rate and Qphred the base quality value of Illumina. This yields a result of Qphred = -10log10(e). The GC content, the proportion of guanine (G) to cytosine (C) in the nucleotide sequence, varies between species. However, the 6 bp random primers employed in reverse transcription cause the initial few bases to have a certain predilection for nucleotide composition, leading to regular fluctuations and subsequent steadiness. The NEB library construction technique requires that, in accordance with random sequence interruption and double-strand complementarity, the GC and AT contents of each position of the sequence read segment be equivalent and remain steady and horizontal throughout the entire sequence. For a chain-specific database, AT or GC separation may occur because only single-chain information is retained. Figure 3B shows the GC content distribution of each sample in this study.

To guarantee the accuracy and dependability of the data analysis, the raw sequencing data were filtered in the following manner: 1) reads with adapters were eliminated; 2) reads with N (the base information was not discernible); and 3) reads of poor quality (Qphred <  = 20 base number comprising more than 50% of the whole read length) were excluded. The filtering of the sequencing data from each sample is shown in Fig. 3C.

After checking the sequencing error rate and the GC content distribution, the original data were filtered, and clean reads were obtained for subsequent analysis. The summary of the data is shown in Table 2.

**Table 2:** The details of the transcriptome assembly result

Sample	Library	Raw _reads	Raw _bases	Clean _reads	Clean _bases	Error _rate(%)	Q20	Q30	GC_pct
C1	Sample 1	45186018	6.78G	43780932	6.57G	0.03	97.65	93.89	58.92
C2	Sample 2	40270380	6.04G	39434346	5.92G	0.03	97.71	93.95	58.89
C3	Sample 3	46773424	7.02G	45533344	6.83G	0.02	98.16	94.57	59.62
C4	Sample 4	42724428	6.41G	41601340	6.24G	0.02	98.20	94.66	56.57
C5	Sample 5	45300782	6.8G	44047466	6.61G	0.03	97.76	93.65	57.94
S1	Sensitivity_1	43300398	6.5G	42447828	6.37G	0.03	97.21	92.33	50.76
S2	Sensitivity_2	39648482	5.95G	38825744	5.82G	0.03	97.11	92.17	51.55
S3	Sensitivity_3	47937238	7.19G	46735142	7.01G	0.03	97.22	92.49	54.72
S4	Sensitivity_4	24241332	3.64G	23893386	3.58G	0.03	96.67	90.94	45.37
E1	Experimental_1	37478390	5.62G	37064522	5.56G	0.03	96.62	91.33	53.36
E2	Experimental_2	29402980	4.41G	29075142	4.36G	0.03	96.75	91.09	44.93
E3	Experimental_3	32223896	4.83G	31400410	4.71G	0.03	96.86	91.28	45.81

### Reference genome alignment

The sum of read1 and read2, that is, the number of clean reads in the data quality summary table, was used to determine the mapping rates of read1 and read2; this is the total read number in the table below. The actual number of reads should be based on the data quality. A summary table (Table 3) shows the proportions of the samples relative to the reference genome.

**Table 3:** Sample and reference genome comparison statistics

Sample	Total reads	Total map	Unique_ map	Multi_map	Read1_map	Read2_map	Positive_map	Negative_map	Exonic	Intronic
C1	43780932	41030531 (93.72%)	26896549 (61.43%)	14133982 (32.28%)	13468455 (30.76%)	13428094 (30.67%)	13431244 (30.68%)	13465305 (30.76%)	97.84%	1.45%
C2	39434346	37476740 (95.04%)	23075521 (58.52%)	14401219 (36.52%)	11561094 (29.32%)	11514427 (29.2%)	11515459 (29.2%)	11560062 (29.31%)	98.03%	1.32%
C3	45533344	43811184 (96.22%)	24330750 (53.44%)	19480434 (42.78%)	12198066 (26.79%)	12132684 (26.65%)	12133320 (26.65%)	12197430 (26.79%)	99.04%	0.64%
C4	41601340	39450866 (94.83%)	28569365 (68.67%)	10881501 (26.16%)	14299447 (34.37%)	14269918 (34.3%)	14245105 (34.24%)	14324260 (34.43%)	98.05%	1.36%
C5	44047466	42090565 (95.56%)	28808366 (65.4%)	13282199 (30.15%)	14470240 (32.85%)	14338126 (32.55%)	14356012 (32.59%)	14452354 (32.81%)	98.39%	1.11%
S1	42447828	39535118 (93.14%)	34962023 (82.36%)	4573095 (10.77%)	17420811 (41.04%)	17541212 (41.32%)	17467140 (41.15%)	17494883 (41.22%)	94.54%	4.17%
S2	38825744	36475226 (93.95%)	35255785 (90.81%)	1219441 (3.14%)	17549530 (45.2%)	17706255 (45.6%)	17608073 (45.35%)	17647712 (45.45%)	86.12%	11.79%
S3	46735142	43343607 (92.74%)	39928429 (85.44%)	3415178 (7.31%)	19897050 (42.57%)	20031379 (42.86%)	19953319 (42.69%)	19975110 (42.74%)	96.32%	2.77%
S4	23893386	21367004 (89.43%)	21173685 (88.62%)	193319 (0.81%)	10572481 (44.25%)	10601204 (44.37%)	10534396 (44.09%)	10639289 (44.53%)	91.73%	4.26%
E1	37064522	31743451 (85.64%)	29091267 (78.49%)	2652184 (7.16%)	14557649 (39.28%)	14533618 (39.21%)	14526902 (39.19%)	14564365 (39.29%)	91.35%	5.85%
E2	29075142	27609750 (94.96%)	27381280 (94.17%)	228470 (0.79%)	13707519 (47.15%)	13673761 (47.03%)	13651029 (46.95%)	13730251 (47.22%)	98.80%	0.44%
E3	31400410	28185248 (89.76%)	27716167 (88.27%)	469081 (1.49%)	13816279 (44.0%)	13899888 (44.27%)	13840701 (44.08%)	13875466 (44.19%)	98.44%	0.64%

According to the comparison results, the proportions of reads in the exon region, intron region and intergene region of the genome were calculated. The general model species had relatively well-annotated genes (e.g., humans and mice), with a high proportion of comparisons to the exon region. Reads matched to intron regions may be derived from precursor mRNAs or introns retained by variable splicing events. Reads in intergenomic regions may be derived from ncRNA or from DNA fragment contamination, or gene annotation may not be perfect. The distribution of sequencing reads in the genome region of all the samples is shown in Fig. 3D.

### Quantitative analysis

The reference genome's gene alignment position information was used to calculate the number of reads from the start to the end of each gene, including the new predicted gene. Reads with alignment quality values less than 10, reads on unpaired alignments, and reads aligned to multiple regions of the genome were filtered out. Subread software10 employs the Counts feature in this analysis [17].

**Fig. 4:**
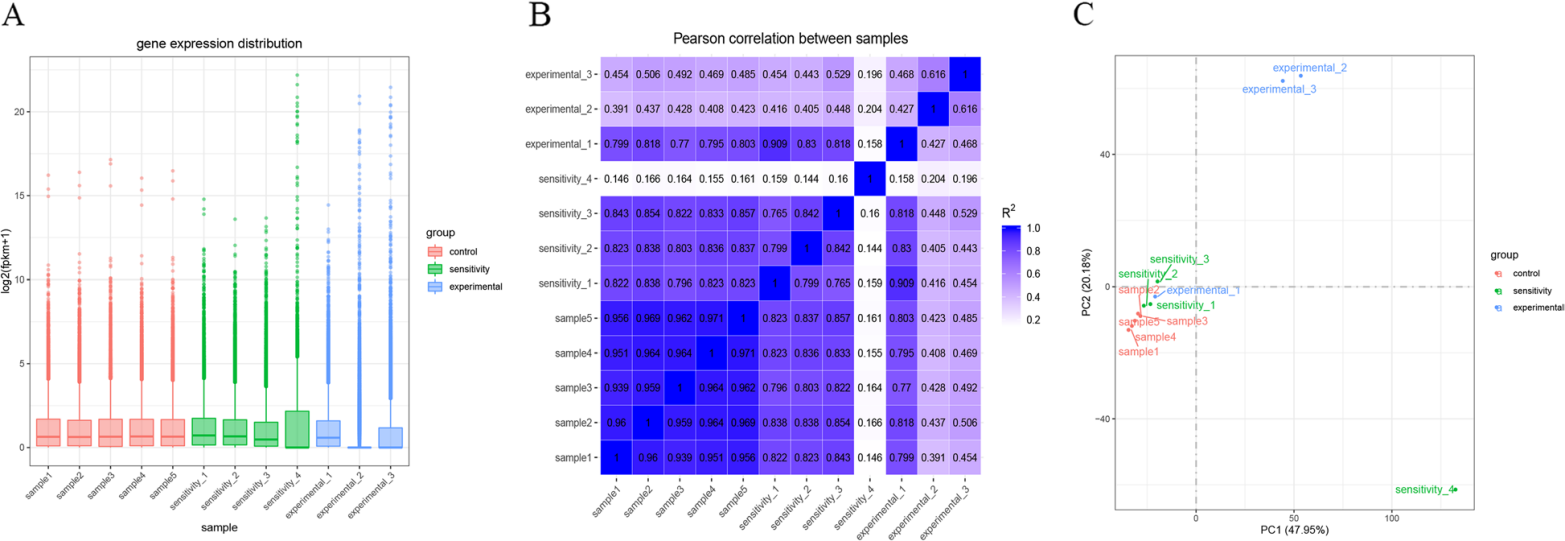
Quantitative analysis of the RNA sequencing samples. **A** Box plot illustrating the distribution of gene expression across samples. **B** Correlation heatmap showing the relationships between samples. **C** The results of principal component analysis (PCA)

We performed quantitative analysis of the gene expression levels for each sample separately and then merged the results to obtain the expression matrix of all the samples [18]. The expression value determined via RNA-seq is not typically determined by read count but rather by FPKM, which has been adjusted for sequencing depth and gene length [19]. After calculating the expression values of all the genes in each sample, box plots (Fig. 4A) were generated to demonstrate the distribution of the gene expression levels in the different samples. The sample name is depicted in the figure. The ordinate is log2(FPKM + 1), and the box plot of each area is composed of five statistics: the maximum, the upper quartile, the median, the lower quartile, and the minimum.

Biological repetition is usually necessary for any biological experiment, and currently, mainstream journals also basically require biological repetition. Biological replication serves two main purposes. One purpose is to demonstrate that the biological experimental manipulations involved are not accidental but reproducible. Another is to ensure more reliable results from subsequent differential genetic analysis. An important measure of the dependability of experiments and the suitability of sample selection is the correlation of gene expression levels between samples. The closer the correlation coefficient is to 1, the more similar the expression patterns are between samples. The Encode program recommends that the square of the Pearson correlation coefficient (R2) should be greater than 0.92 under ideal sampling and experimental conditions. The R2 between biological replicates in this project operation must be at least 0.8; otherwise, the sample must be properly interpreted, and the experiment must be repeated. The FPKM values of all genes in each sample were used to calculate the correlation coefficients of samples within and between groups, which can be visualized in a heatmap to show the sample differences between groups and the repetition of samples within a group. When the correlation coefficient between samples is greater, their expression patterns become more closely aligned. The sample correlation heatmap is shown in Fig. 4B.

The figure below displays the gene expression values (FPKM) of all samples that underwent principal component analysis (PCA), a process that utilizes linear algebra calculations to reduce dimensionality and extract principal components for tens of thousands of genetic variables. This technique is also frequently employed to assess the distinctions between groups and the repetition of samples within a group. It is desirable that the PCA plot have samples between groups dispersed, and samples within groups clustered together. The principal component analysis results are shown in Fig. 4C.

### Analysis of DEGs

After the quantification of gene expression is completed, statistical analysis of the expression data is required to screen genes with significantly different expression levels in the samples under different states. Difference analysis can be divided into three steps. The original read count was first normalized, mainly to correct the sequencing depth. The statistical model calculates the hypothesis test probability (p value) and finally performs multiple hypothesis test correction to obtain the FDR value (false discovery rate, padj is its common form) [20, 21].

From the quantification of gene expression in our research, we found that two samples, experimental 1 and sensitivity 4, were not suitable for clinical behavior. Therefore, in the subsequent process of data analysis, the two samples were separated from the other 5 patient samples.

In general, if a gene has a more than two fold difference in expression between two groups of samples, we consider that the gene is differentially expressed. To judge whether the difference in expression between the two samples was due to various errors or an essential difference, we needed to perform hypothesis testing on the expression data of all the genes in the two samples. Transcriptome analysis is performed on thousands of genes, which can lead to the accumulation of false positives. The *p* value of the hypothesis test was corrected by introducing padj, thus controlling the proportion of false positives16. This is because a greater number of genes leads to a greater accumulation of false positives [22].

**Fig. 5:**
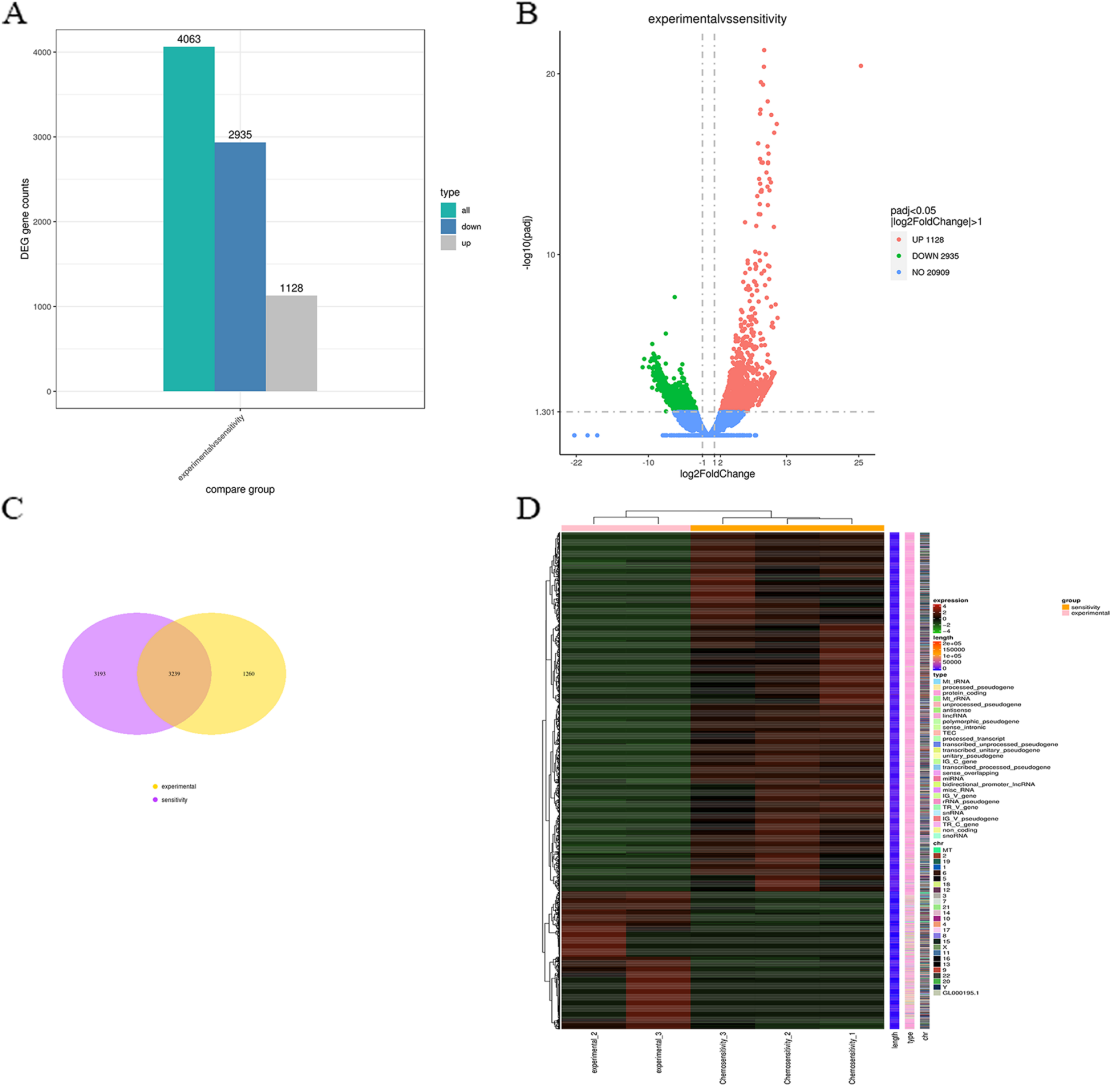
Difference analysis between chemotherapy sensitivity and chemotherapy resistance PTCL samples. **A** The bar chart depicting the number of DEGs identified in the various comparisons. **B** The volcano plot depicting differential gene expression. **C** The venn diagram depicting the overlap of differentially expressed genes. **D** The clustering heatmap of the differentially expressed genes

The screening criteria for differentially expressed genes are highly important. The criteria |log2(fold change)|> = 1 and padj <  = 0.05 are commonly used empirical values that can be flexibly selected according to the actual project. Figure 5A shows the statistics for the number of DEGs (both up- and downregulated) for each comparison combination.

A volcano map was constructed to visualize the distribution of DEGs associated with each comparative combination, as shown in Fig. 5B. In the figure, the x-coordinate indicates the change in the gene expression ratio between the treatment and control groups (log2FoldChange), and the y-coordinate indicates the level of significance of the difference in gene expression between the treatment and control groups (-log10padj or -log10*p*value). The red dots represent upregulated genes, and the green dots represent downregulated genes.

A venn diagram can illustrate the common or exclusive differential genes identified in certain comparison combinations, as well as the overlap of differential genes between them. The total number of DEGs in the comparison combination is represented by the sum of all the genes in the circle of the Venn diagram, while the overlap area is the number of DEGs common to the combinations (Fig. 5C).

The differentially expressed genes of all the comparison groups were merged to form the difference gene set. Cluster analysis of diverse gene sets can be performed for more than two experiments, and genes with analogous expression patterns can be amalgamated. We used mainstream hierarchical clustering to perform cluster analysis on the FPKM values of genes and normalize the rows (Z scores). Clustering genes or samples with similar expression patterns in the heatmap, the color in each square does not reflect the gene expression value but rather the value obtained after the expression data rows are normalized (usually between -2 and 2). Therefore, the colors in the heatmap can be compared only horizontally, representing the expression of the same gene in different samples, and vertically, representing the expression of different genes in the same sample. There are both clusters between groups and clusters between samples in the results file. Figure 5D shows the clustering of samples in the conclusion report.

### Enrichment analysis

After we obtained DEGs based on gene expression analysis, we further examined the functions of the genes. For transcriptome analysis, thousands of genes are often involved, which complicates the analysis. The solution is to divide a gene list into multiple parts, thereby reducing the complexity of the analysis. Gene function enrichment analysis is typically employed to ascertain how to separate genes into distinct groups, with the aim of uncovering biological pathways that are essential for biological operations, thereby revealing and comprehending the fundamental molecular processes of biological operations. Functional enrichment analysis can classify hundreds of genes, proteins or other molecules into different pathways to reduce the complexity of the analysis. In addition, the activated pathways were clearly more convincing than a simple list of genes or proteins under two different experimental conditions. The construction of a gene set (e.g., GO and KEGG databases) for classification is the initial step in gene function enrichment analysis [23, 24]. The next step involved mapping our target gene set (differential or otherwise) to the background gene set, with a focus on distinguishing between annotation and enrichment.

We used ClusterProfiler software to determine the enrichment of GO functions and KEGG pathways in the diverse gene sets. Hypergeometric distribution serves as the basis for the enrichment analysis, wherein the differential gene set is the differential gene set obtained from the differential analysis and annotated to the GO or KEGG database, and the background gene set is all the genes that have undergone significant differential analysis and annotated to the GO or KEGG database. Enrichment analysis revealed enrichment of all the DEGs, as well as upregulation of the DEGs and downregulation of the DEGs for each differential comparison combination. The table shown in this report is the enrichment analysis result of a selected comparison combination, and the picture is the enrichment analysis result of all combinations.

**Fig. 6:**
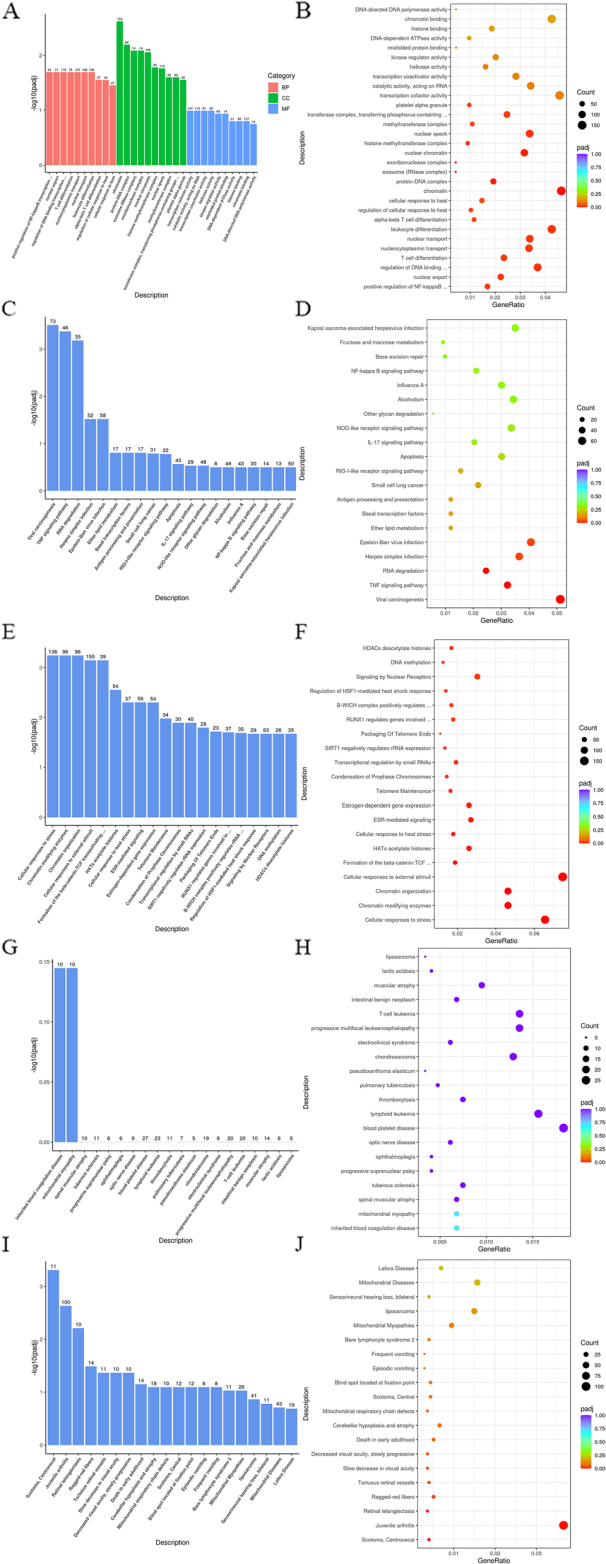
Enrichment analysis between chemotherapy sensitivity and chemotherapy resistance PTCL samples. **A** The GO enrichment analysis bar diagram. **B** The GO enrichment analysis scatter plot. **C** The KEGG enrichment analysis bar diagram. **D** The KEGG enrichment analysis scatter plot. **E** The Reactome enrichment analysis bar diagram. **F** The Reactome enrichment analysis scatter plot. **G** The DO enrichment analysis bar diagram. **H** The DO enrichment analysis scatter plot. **I** The DisGeNET enrichment analysis bar diagram. (J)The DisGeNET enrichment analysis scatter plot

A comprehensive database, GO (Gene Ontology), which can be divided into three parts—biological process (BP), cellular component (CC), and molecular function (MF)—was used to describe gene function. To achieve significant enrichment, padj must be lower than 0.05 for GO functional enrichment. The 30 most significant terms from the GO enrichment analysis were subsequently chosen for bar charts, with all the terms being drawn if there were fewer than 30 terms, as shown in the figure below. The figure displays the GO terms in the horizontal coordinate and the significance level of enrichment in the vertical coordinate. The higher the value is, the more significant the difference is. The colors in the figure (Fig. 6A) represent the three GO subclasses of BP, CC and MF. Scatter plots of the 30 most significant terms from the GO enrichment analysis were generated; if fewer than 30 terms were present, all the terms were included, as illustrated in the figure (Fig. 6B).

The Kyoto Encyclopedia of Genes and Genomes (KEGG) is a vast repository of genomic, chemical, and systemic functional information. For KEGG pathway enrichment, padj was less than 0.05 as the threshold for significant enrichment. The enrichment results are shown in Fig. 6C.

The significantly downregulated and upregulated (experiment vs sensitivity) KEGG pathways are listed in the table (Tables 4, 5). Scatter plots were created from the KEGG enrichment results, with the most noteworthy 20 pathways chosen. For genes with fewer than 20 KEGG pathways, all pathways were plotted, as shown in the figure below. The ratio of DEGs annotated to the KEGG pathway to the total number of DEGs is depicted in the figure as the horizontal coordinate, the KEGG pathway is represented by the vertical coordinate, dots signify the number of genes annotated to the KEGG pathway, and the color from red to purple symbolizes the importance of enrichment (Fig. 6D).

**Table 4:** Significant down regulated (Experiment vs Sensitivity) KEGG

KEGGID	Description	GeneRatio	BgRatio	*P* value	padj
hsa03018	RNA degradation	34/1136	71/5752	7.38E-08	2.29E-05
hsa04668	TNF signaling pathway	42/1136	100/5752	2.37E-07	3.68E-05
hsa05169	Epstein-Barr virus infection	55/1136	158/5752	4.99E-06	0.000515
hsa05168	Herpes simplex infection	46/1136	138/5752	9.97E-05	0.007724
hsa04612	Antigen processing and presentation	17/1136	37/5752	0.000267	0.016567
hsa04622	RIG-I-like receptor signaling pathway	21/1136	53/5752	0.000645	0.033322

**Table 5:** Significant up regulated (Experiment vs Sensitivity) KEGG

KEGGID	Description	GeneRatio	BgRatio	*P* value	padj
hsa05034	Alcoholism	32/290	150/5752	1.28E-12	3.33E-10
hsa05322	Systemic lupus erythematosus	25/290	95/5752	3.03E-12	3.96E-10
hsa04611	Platelet activation	18/290	108/5752	5.88E-06	0.000512
hsa05203	Viral carcinogenesis	23/290	178/5752	2.51E-05	0.001638
hsa04610	Complement and coagulation cascades	11/290	56/5752	8.62E-05	0.004189
hsa05012	Parkinson disease	18/290	132/5752	9.63E-05	0.004189
hsa00190	Oxidative phosphorylation	16/290	120/5752	0.000306	0.011402
hsa04723	Retrograde endocannabinoid signaling	15/290	114/5752	0.000543	0.017713
hsa04810	Regulation of actin cytoskeleton	20/290	182/5752	0.000764	0.022168

The Reactome database aggregates responses and biological pathways in model species such as humans. Padj values less than 0.05 were used as the threshold for significant enrichment in the Reactome pathway, and the enrichment results are shown in Fig. 6E.

The scatter plots for display, as depicted in the figure (Fig. 6F), were generated from the analysis of Reactome enrichment, with the most significant 20 pathways chosen; if fewer than 20 were present, all pathways were included. Disease Ontology (DO) is a database that describes human gene functions related to diseases. For DO enrichment, a padj value less than 0.05 was used as the threshold for significant enrichment, and the enrichment results are shown in the figure below (Fig. 6G). The scatter plots for display were chosen from the DO enrichment analysis, with the 20 most significant terms chosen; any terms that were less than 20 were excluded, as depicted in the figure (Fig. 6H).

The DisGeNET database integrates human disease-related genes. The threshold for significant enrichment in DisGeNET enrichment was a padj value less than 0.05, and the enrichment results are depicted in the figure below (Fig. 6I).

Figure 6J shows the selection of the 20 most significant terms from the DisGeNET enrichment analysis for bar charts; any terms below this amount were excluded, and all the terms were included. In the figure, the horizontal coordinate is the DisGeNET term, and the vertical coordinate is the enrichment significance level of the term. The significance increases proportionally to the magnitude of the value.

To elucidate the potential pathogenesis of peripheral T-cell lymphoma chemotherapy resistance, a comprehensive analysis of differentially expressed genes (DEGs) was conducted, including Gene Ontology (GO) enrichment, Kyoto Encyclopedia of Genes and Genomes (KEGG), Reactome enrichment, Disease Ontology (DO), and DisGeNET enrichment. These signaling pathways may serve as potential novel molecular targets for therapy [25].

**Fig. 7:**
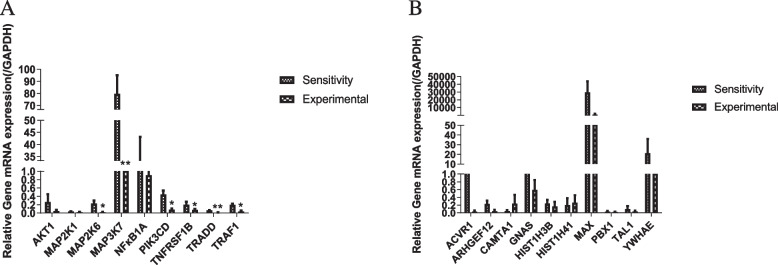
Verification of the accuracy of the differentially expressed genes (DEGs) between chemotherapy sensitivity and chemotherapy resistance PTCL samples. **A** The chart shows the RT‒qPCR analysis results for the genes whose expression was downregulated in chemotherapy resistance PTCL samples. *P < 0.05,**P < 0.01. **B** The chart shows the RT‒qPCR analysis results for the genes whose expression was downregulated in chemotherapy resistance PTCL samples

To verify the accuracy of the DEGs, 9 downregulated DEGs (*AKT1, NFKB1A, TRADD, MAP2K1, MAP2K6, MAP3K7, PIK3CD, TRAF1, TNFRSF1B*) and 10 up regulated DEGs (*CAMTA1, HIST1H3B, ARHGEF12, PBX1, HIST1H4I, TAL1, YWHAE, ACVR1, MAX, GNAS*) were confirmed in a quantitative real time PCR comparison of chemotherapy resistance in extranodal peripheral T cell lymphoma from 6 clinical specimens. The sequences of primers used are listed in Table 6. As shown in Fig. 7A, there were significant differences in the down regulated DEGs, while the up regulated DEGs didn’t show significant differences (Fig. 7B).

**Table 6:** Real-Time PCR Primer Sequences List

Genes Name	Primers	Sequence(5’ → 3’)
NFKBIA	NFKBIA Forward Primer	CCCGCACCTCCACTCCATCC
	NFKBIA Reverse Primer	AGCATTGACATCAGCACCCAAGG
AKT1	AKT1 Forward Primer	GCAGGATGTGGACCAACGTGAG
	AKT1 Reverse Primer	GCAGGCAGCGGATGATGAAGG
PIK3CD	PIK3CD Forward Primer	CAGGTGAACGGCAGGCATGAG
	PIK3CD Reverse Primer	TGGCGAGGATGGAGGAGGAATG
TRAF1	TRAF1 Forward Primer	TTGGAGCAGAGGGTGGTGGAG
	TRAF1 Reverse Primer	CCGCCTGGTGACATTGGTGATC
TNFRSF1B	TNFRSF1B Forward Primer	CACGCAGCCAACTCCAGAACC
	TNFRSF1B Reverse Primer	AGTCGCCAGTGCTCCCTTCAG
MAP2K1	MAP2K1 Forward Primer	TCATCTGGAGATCAAACCCGCAATC
	MAP2K1 Reverse Primer	CCATCGCTGTAGAACGCACCATAG
MAP2K6	MAP2K6 Forward Primer	GCTCAACCAGAAGGGATACAGTGTG
	MAP2K6 Reverse Primer	TGTGGCGATGGCTCCTCTACC
MAP3K7	MAP3K7 Forward Primer	GCAACCACAGGCAACGGACAG
	MAP3K7 Reverse Primer	ACACTGGGACTGGATGACCTACTG
TRADD	TRADD Forward Primer	CGCCACCTGCCCAGACTTTTC
	TRADD Reverse Primer	CGCCATTTGAGACCCACAGAGC
GNAS	GNAS Forward Primer	GCCTGCTACGAACGCTCCAAC
	GNAS Reverse Primer	TCCTGATCGCTCGGCACATAGTC
MAX	MAX Forward Primer	GACGCTGACAAACGGGCTCATC
	MAX Reverse Primer	AGCTTCTCTCCTTGGAGTGATGGG
ACVR1	ACVR1 Forward Primer	CGAAGGGCTCATCACCACCAATG
	ACVR1 Reverse Primer	CCTTTCCCGACACACTCCAACAG
YWHAE	YWHAE Forward Primer	AGGAAGGAGGCTGCGGAGAAC
	YWHAE Reverse Primer	ATAGGATGCGTTGGTGGAAGTTCTG
TAL1	TAL1 Forward Primer	GCCTTCCCTATGTTCACCACCAAC
	TAL1 Reverse Primer	TTCACATTCTGCTGCCGCCATC
HIST1H4I	HIST1H4I Forward Primer	CTACACGGAGCACGCCAAGC
	HIST1H4I Reverse Primer	TAGCCGCCGAAGCCATAGAGG
PBX1	PBX1 Forward Primer	GCGGTGATGATCCTGCGTTCC
	PBX1 Reverse Primer	CTTGGCTAACTCCTCTTTGGCTTCC
ARHGEF12	ARHGEF12 Forward Primer	TGCCTGCCAATTCCATGTCTTCTG
	ARHGEF12 Reverse Primer	AGGTGTGCCATCTAAGGTGTCTCC
HIST1H3B	HIST1H3B Forward Primer	AGAAATCGCCCAAGACTTCAAGACC
	HIST1H3B Reverse Primer	GTTTGTGTCCTCAAAGAGCCCTACC
CAMTA1	CAMTA1 Forward Primer	AGGGGAAATGGCTGCCGAAAAC
	CAMTA1 Reverse Primer	CTTCTATAGGTGGCACGGTGTTGAG

### Fusion gene analysis

In this study, fusion genes were detected using STAR-Fusion software. Through this analysis, we identified a number of fusion genes in the sample groups under investigation. A detailed list of these fusion genes is presented in Table 7. The detection of fusion genes provides insights into potential oncogenic drivers and can contribute to a better understanding of the underlying molecular mechanisms involved in the development and progression of the specific disease or condition being investigated. This knowledge provides a foundation for further investigation into the biological and clinical implications of these fusion events in the context of the studied disease or condition.

**Table 7:** Fusion Gene list of the RNA Sequence Result

Groups	Sample Name	Fusion Genes List				
Corntrol	Sample1	RSRP1–TMEM50A	AL359644.1–GGTA1P	XRCC5–STAT1		
	Sample2	HNRNPUL1–BCL6	PFKFB3–LINC02649	RBM38–UBB	TSPAN5–UBB	YAF2–KDM3B
	Sample3	HBA1–B2M				
	Sample4	LINC00402–AL353660.1				
	Sample5	NCEH1–SPATA16	IGH@-ext–PRPF8			
Sensitivity	Sensitivity-1	/				
	Sensitivity-2	AC005280.2–NUMB	ANKRD11–MAP1LC3B	B3GNT5–BCL6	B3GNT5–KLHL24	DDX5–ATP6V0C
		EEF1D–ING1	EEF1D–ZC3H3	ELOVL5–PNRC1	FBXL5–PNRC1	GLT1D1–ETS2
		GLT1D1–PFKFB3	HNRNPK–NFIL3	HRH2–ING1	IER2–KLF2	ITGAX–FES
		IVNS1ABP–PNRC1	LATS2–ING1	LBR–H3-3A	LENG8–CNOT3	LITAF–SNN
		LRCH4–ATP6V0C	LRCH4–KLF2	LRCH4–TMC6	LSP1–PNRC1	MBP–ITGB2
		MIDN–KLF16	MYO9B–KLF2	NAMPT–FKBP8	NAMPT–SLC25A37	NINJ1–NFIL3
		OAZ1–KLF2	PFKFB3–EEF1D	PFKFB3–LINC02649	PFKFB3–UBB	PGS1–DDX5
		PGS1–LINC01578	PIM3–CFL1	PIM3–RAC2	PLXNB2–SCO2	POU2F1–GLUL
		RAB20–ING1	SBNO2–KLF16	SEPTIN14–AP006222.1	SH2B2–ING1	SLC19A1–ITGB2
		SLC66A2–CTDP1	SLC7A5–LINC01578	ST3GAL1–GLUL	TAGLN2–PHC2	TMCC3–MAEA
		TMCC3–UBC	UBE2J1–PNRC1	WDR1–MAEA	YPEL5–ANP32A	YPEL5–PHC2
		YWHAE–DDX5	ZC3H3–CTDSP1	ZNF292–PNRC1		
	Sensitivity-3	ARRDC2–KLF2	C19orf25–DAZAP1	LILRA2–AC245884.12	PLXNB2–SCO2	SBF1–SCO2
		SLC16A3–ATP6V0C	TRABD–SCO2	ZEB2–PTMA		
	Sensitivity-4	/				
Experimental	Experimental_1	AC020916.1–KLF2	CSNK1G2–KLF16	NOTCH1–ATP6V0C	ARF1–H3-3A	BAZ1A–GNAS
		ELL–KLF2	FRY–RHEB	GNB2–RNF166	NFIL3–HNRNPK	NINJ1–METRNL
		NOL4L–UBC	NOP53–DHX34	RABGEF1–MFSD12	RENBP–S100A9	YPEL2–ACSL1
	Experimental_2	/				
	Experimental_3	/				

## Discussion

Peripheral T-cell lymphoma (PTCL) is a rare subtype of T-cell lymphoma characterized by aggressive clinical behavior and relative resistance to chemotherapy. Understanding the underlying mechanisms responsible for the development of chemotherapy resistance in PTCL patients is crucial for advancing our knowledge in this area. The study of abnormally expressed genes in the context of chemotherapy resistance in PTCL patients is an important step toward unraveling the complex mechanisms underlying this disease and refining our strategies for its diagnosis, treatment and management [26].

Resistance to therapy in peripheral T-cell lymphoma (PTCL) can be influenced by the activation status of lymphomatocytes [27, 28]. The activation state of lymphomatoblasts has the potential to impact their response to antitumor drugs, thereby affecting the effectiveness of chemotherapy. If lymphoblasts remain in a continuously activated state, they may develop resistance to chemotherapy drugs, leading to ineffective treatment outcomes. Hence, understanding the activation status of lymphomatoblasts is crucial in guiding physicians to select the most suitable treatment plan, ultimately improving the efficacy of chemotherapy.

Furthermore, resistance to therapy in peripheral T-cell lymphoma (PTCL) can also be attributed to the immature development of T-cell tumors [29]. The development and maturation of T-cell tumors are regulated by various factors, including gene mutations, abnormal signal transduction pathways, and epigenetic modifications. These abnormalities can lead to abnormal proliferation and malignant transformation of T-cell tumors while also impacting their response to therapy. During treatment, certain T-cell tumors may develop resistance, resulting in the failure of chemotherapy or targeted therapy. This resistance can be linked to the immature development of T-cell tumors. Studies have suggested that immature T-cell tumors may have a greater propensity for genetic mutations and abnormal signal transduction pathways, rendering them resistant to treatment [30].

The content of transcriptome analysis includes data quality control, gene structure analysis and gene expression level analysis, the core of which is gene expression level analysis.Transcriptome data mining basically follows a global-to-local approach and can analyze the expression levels, functions, and specific genes of a gene [31].

Generally, our experimental designs are based on phenotypes, and the changes at the gene level obtained through transcriptome data reflect the differences in phenotypes. Differentially expressed gene (DEG) screening is the core basis of transcriptome sequencing analysis. The genes with high expression and great differences, or common differential genes, are often the genes we can focus on.

Genes involved in the same biological process are usually controlled by the same regulatory system; that is, genes involved in the same biological process have similar or the same change rules. By examining the expression of genes in diverse samples, gene modules with comparable expression patterns can be identified, and the most significant gene modules can be identified by phenotype. Common gene expression pattern analysis techniques include cluster heatmaping, trend analysis, and weighted correlation network analysis (WGCNA) [32, 33]. Cluster heatmap analysis was suitable for analysis after certain genes were initially identified. Trend analysis requires an experimental design with at least 3 continuous variables. WGCNA is suitable for experimental designs with large sample sizes. Either way, the goal is to find the gene with the expression module most related to the trait, and the module with the expression pattern consistent with or opposite to the trait change should be the focus.

Genes whose expression levels are consistent must eventually return to their functions. Enrichment analysis was used to classify the functions of genes through different databases. The purpose of enrichment analysis is to study the pathways in which DEGs are significantly enriched. The pathways that were significantly enriched according to the enrichment analysis were definitely key pathways that play important roles, and these pathways were directly discussed and analyzed. In addition, we can directly identify the pathway of interest according to the research purpose. For example, when we study the growth and development of plants, the Plant Hormone Signal transduction pathway must be the focus of research [34].

We identified several genes that may play a role through the above ideas. These genes are called potential key genes. The next step was to further analyze these genes and target genes. We can analyze the expression level and function of the candidate genes. The key genes must have high expression levels and significant differences and must have functions consistent with our research purposes. A common idea of quickly targeting genes is to look for "star" molecules, which requires literature collection, summarizing certain star genes from previous studies, and then focusing on the expression of these genes in our project and structural information [35].

Assuming that our purpose is to study cancer treatment efficacy, we must pay attention to anticancer genes such as P53. On the other hand, in addition to the gene level, we can also focus on pathways, such as pathways related to cancer and other cancer-related signaling pathways. Another way is that we can make stars ourselves. If some of the key genes we found were not star genes, we could find ways to rely on research hotspots to make these new genes stars, such as transcription factor (TF) research.

Our findings indicated that the expression of different genes, particularly TNFRSF1B, TRADD, TRAF1, PIK3CD, MAP3K7, and MAP2K6, may be important for chemotherapy resistance in peripheral TCL. The TNF receptor superfamily member 1B (TNFRSF1B), TNFRSF1A linked by the death domain TRADD, and TRAF1 associated with TNF receptors are inhibited in peripheral T-cell lymphoma when chemotherapy resistance occurs. In addition, phosphatidylinositol-4,5-bisphosphate 3-kinase catalytic subunit delta (PIK3CD), mitogen-activated protein kinase kinase kinase 7 (MAP3K7) and mitogen-activated protein kinase kinase 6 (MAP2K6) are downregulated in peripheral T-cell lymphoma during chemotherapy resistance. These downregulated genes may be potential molecular biomarkers for diagnosing chemotherapy resistance in peripheral T-cell lymphoma patients.

The expression data of various genes revealed that the TNF signaling pathway, NF-kappa B signaling pathway, apoptosis signaling pathway, AMPK signaling pathway, Epstein–Barr virus infection pathway, and Herpes simplex infection were the primary signaling pathways likely responsible for the emergence of chemotherapy resistance in peripheral T-cell lymphoma. The TNF-α and NF-kappa B pathways are closely linked to tumor progression and resistance to treatment [36]. In the Epstein–Barr virus infection signaling pathway, Herpes simplex infection is the main cause of peripheral T-cell lymphoma [37, 38]. The PI3K/AKT/MAPK and NF-kappa B signaling pathways, in response to external signals, encourage metabolism, proliferation, cell survival, growth, and angiogenesis [39]. AMP-activated protein kinase (AMPK) is a major factor in energy balance, thus protecting cells from detrimental pressures through the coordination of multiple metabolic pathways. Notably, AMPK activation was recently shown to mediate metabolic reprogramming in drug-resistant cancer cells [40]. By targeting these signaling pathways, drug resistance to peripheral T-cell lymphoma therapy may be overcome.

To conclude, this research has enhanced our understanding of the link between signaling pathways and chemotherapy resistance in peripheral T-cell lymphoma, yet certain limitations remain. First, although we investigated the association between signaling pathways and chemotherapy resistance in peripheral T-cell lymphoma patients, there is a dearth of clinical patients for whom the strong connection between them has been confirmed; therefore, we still need to increase the number of patients to verify our findings.

Second, we found that there were no significant changes in the up regulated DEGs, such as *CAMTA1, HIST1H3B, ARHGEF12, PBX1, HIST1H4I, TAL1,YWHAE,ACVR1,MAX,GNAS*. Therefore, investigation the up regulated DEGs of resistance to chemotherapy in patients with peripheral T-cell lymphoma must be pursued in additional patient studies.

Third, the concentration of primary lymphocytes is low in the selected peripheral T-cell lymphoma samples. Therefore, we still need to carry out RNA sequencing experiments on additional primary lymphocyte samples.

## Conclusion

Understanding the changes in gene expression and signaling pathways associated with chemotherapy resistance in PTCL patients could help identify novel therapeutic targets and develop personalized treatment strategies. By targeting specific genes or signaling pathways that are upregulated or downregulated during chemotherapy resistance, it may be possible to overcome drug resistance and improve patient outcomes. RNA sequencing provides a comprehensive and detailed analysis of the transcriptome, allowing for a deeper understanding of the molecular mechanisms underlying chemotherapy resistance in PTCL. This groundbreaking discovery suggests that the expression patterns of multiple genes, particularly TRADD2 and MAP3K7, may play a crucial role in chemotherapy resistance in peripheral T-cell lymphoma (PTCL). Furthermore, the TNF signaling pathway has been identified as potentially involved in the development of resistance to chemotherapy in PTCL. In summary, these various genes and signaling pathways with altered expression could serve as promising prognostic factors. Their potential utilization in clinical practice has the potential to aid in the identification of more effective treatment strategies for multidrug resistance in PTCL. By considering the expression profiles of these genes and understanding their association with specific signaling pathways, clinicians can make informed decisions to tailor treatment plans and improve patient outcomes in patients with PTCL harboring chemotherapy resistance.

## Data Availability

The datasets generated in this study are available in the online NCBI GEO repositories at the following link: https://www.ncbi.nlm.nih.gov/geo/query/acc.cgi?acc=GSE250050. The hyperlinking procedure for the software packages ClusterProfiler and DeSeq2 are as follows: DeSeq2: https://bioconductor.org/packages/release/bioc/html/DESeq2.html ClusterProfiler: http://bioconductor.org/packages/release/bioc/html/clusterProfiler.html
